# Recurrent Primary Suprahepatic Abscess Due to Providencia Stuartii: A Rare Phenomenon

**DOI:** 10.7759/cureus.1691

**Published:** 2017-09-16

**Authors:** Kyawzaw Lin, Aung Naing Lin, Sandar Linn, Madhavi Reddy, Anjali Bakshi

**Affiliations:** 1 Medicine, The Brooklyn Hospital Center; 2 Internal Medicine, The Brooklyn Hospital Center; 3 GI Department, The Brooklyn Hospital Center; 4 Infectious Disease Department, The Brooklyn Hospital Center

**Keywords:** hepatology, rare infection, pyogenic liver abscess, providencia stuartii, gastroenterology

## Abstract

Gram-negative urease-producing bacilli, Providencia stuartii (P. stuartii), is reported in urinary tract infections, gastroenteritis, and bacteremia in humans but they rarely present with a hepatic abscess. We present a rare case of a recurrent suprahepatic cyst due to P. stuartii in a 45-year-old female, intravenous ( IV) heroin abuser with chronic hepatitis B and C.

A 45-year-old female with 10 days status post right suprahepatic abscess drainage presented with recurrent, right, upper quadrant abdominal pain for one day. The pain was 7/10, sharp, radiated to the right back, and was associated with nausea, non-bloody non-bilious vomiting, and right-sided pleuritic chest pain. She was discharged after interventional radiology (IR) drainage of the abscess and completed 14 days of levofloxacin and metronidazole. On palpation, mild tender hepatomegaly was noticed. Complete blood count showed leukocytosis of 17.1 with left shift but liver enzymes within normal limits. Aspirated fluid cultures from the abscess showed P. stuartii. Blood and urine cultures were negative. A human immunodeficiency virus (HIV) test was negative. Hepatitis B virus (HBV) deoxyribonucleic (DNA) polymerase chain reaction (PCR) showed > 17 million IU/ml and hepatitis C virus (HCV) Ab reactive. A right, upper quadrant sonogram showed 4.1x0.9x2.7 cm fluid collection anterior to the right liver lobe. A computed tomography (CT) abdomen showed a dominant 5.2x5.5x3.9 cm hypodense lesion consistent with an abscess above the right liver. Initially, she was treated empirically with IV piperacillin-tazobactam and anticoagulation for a pyogenic liver abscess (PLA). Clinical and laboratory improvement were achieved with intravenous antibiotics evidenced by the decreasing size of the abscess on repeat CT scan. The patient was discharged with continuing antibiotics after four weeks. Repeated CT scan showed complete resolving of the suprahepatic cyst.

In conclusion, in our patient, long-term shelter residence, IV heroin use, and chronic hepatitis B and C might be precipitating factors for PLA. Managing a recurrent primary hepatic abscess caused by P. stuartii is similar to PLA from other bacterial causes: drainage and antibiotic therapy. However, in our case, she responded well to medical treatment without further surgical drainage.

## Introduction

The gram-negative urease-producing bacilli, Providencia stuartii (P. stuartii), is reported in a variety of infections of the urinary tract, gastroenteritis, and bacteremia in humans. P. stuartii is an opportunistic organism and is usually associated with severe burns, chronic urinary catheterization, and in patients from nursing homes or assistant facilities. The organism is usually isolated from human secretions, including urine, sputum, blood, stool, and wound cultures. However, septicemia secondary to P. stuartii is from the urinary source. The treatment of choice is based on antibiotic sensitivities, the source of infection, and comorbid conditions. A Providencia infection is rare and nosocomial - bacteria pericarditis in one case report - while P. rettgeri is a causal agent in ocular infections - keratitis, conjunctivitis, and endophthalmitis in another case [1-2]. However, rarely have such patients presented with a hepatic abscess as a causal agent. The prevalence of P. stuartii is on the rise due to an antibiotic resistance secondary to extended beta-lactamase (ESBL) enzymes. We present a rare case of a recurrent suprahepatic cyst due to P. stuartii in a 45-year-old female presenting with severe abdominal pain, nausea, and vomiting 10 days after interventional radiology (IR) -guided right suprahepatic cyst drainage under appropriate antibiotics. Later, IR-guided fluid cultures showed P. stuartii, which responded well to piperacillin and tazobactam without further surgical drainage.

## Case presentation

**Figure 1:**
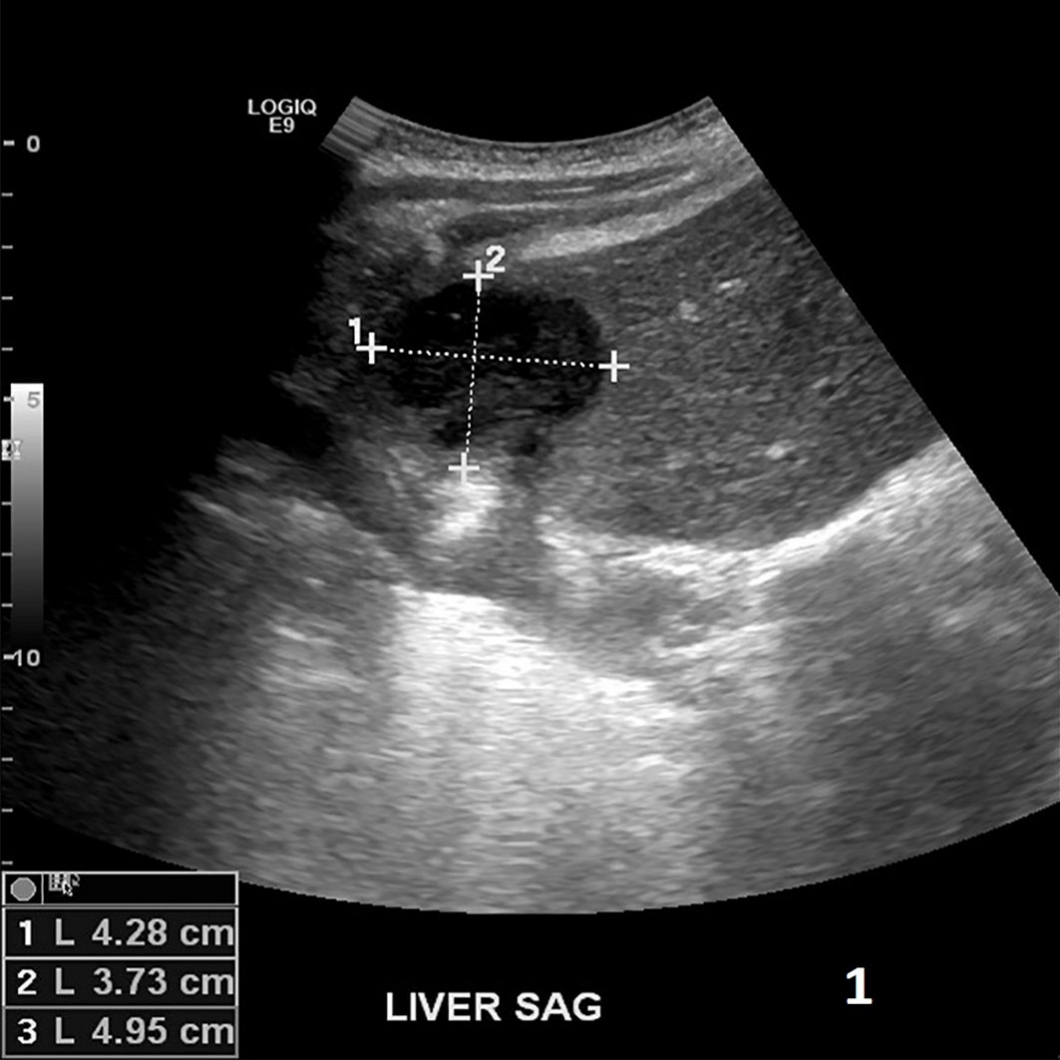
Right upper quadrant sonogram showed 4.1x0.9x2.7 cm cystic formation/well-defined fluid collection in the right upper abdomen anterior to the right liver lobe

**Figure 2:**
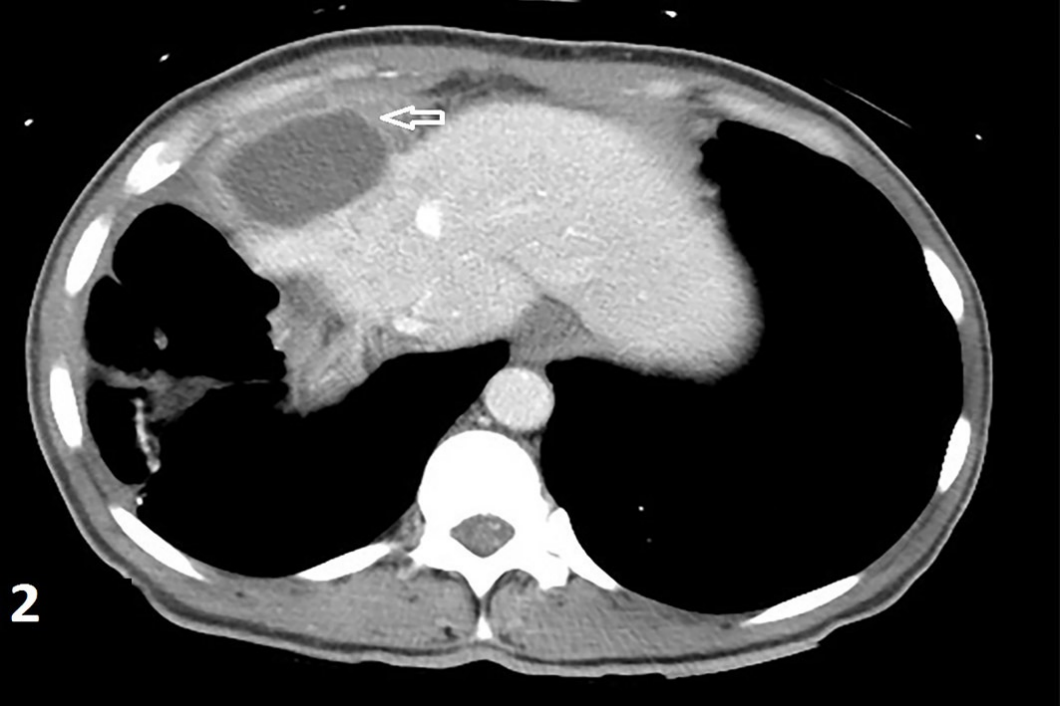
Computed tomography scan (abdomen) showed a dominant 5.2x5.5x3.9 cm hypodense lesion consistent with an abscess above the right liver

**Figure 3:**
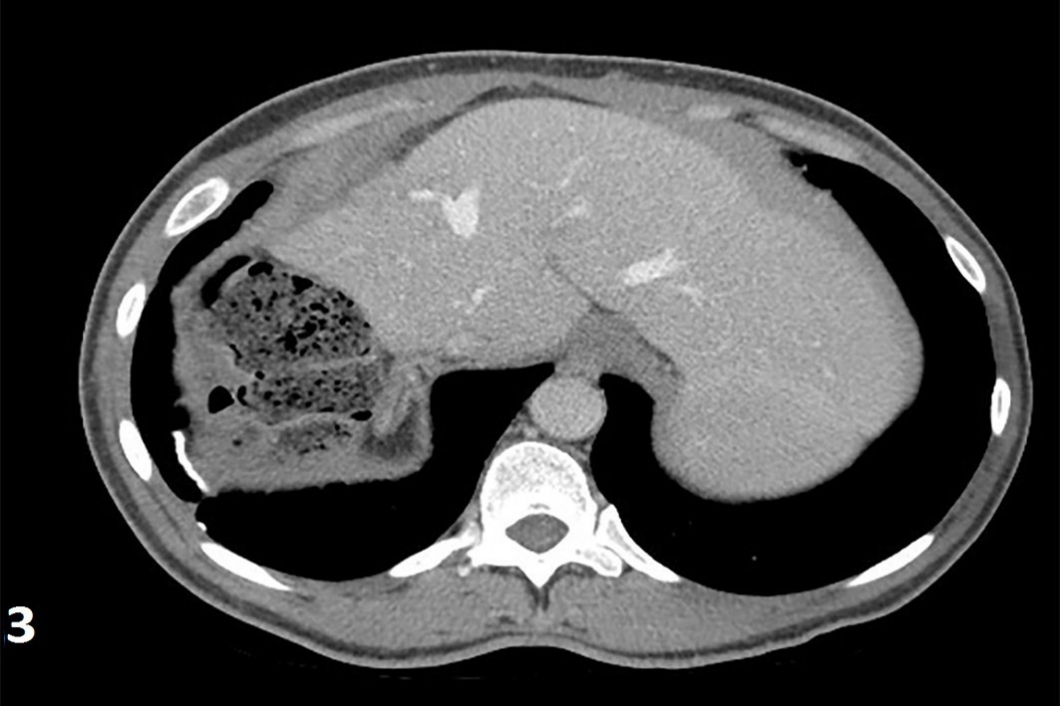
Computed tomography scan (abdomen) (after four weeks of antibiotics) showed complete resolving of the previously seen small collection/cyst anterior to the liver dome since the previous exam. No evidence of liver mass or collection. No evidence of perihepatic fluid or collection.

A 45-year-old female with 10 days status post right suprahepatic abscess drainage and a significant past medical history of chronic hepatitis B and C presented with a recurrent right upper quadrant abdominal pain for one day. She stated that the pain was 7/10, sharp, radiated to the right back, and was associated with nausea, non-bloody, non-bilious vomiting, and right-sided pleuritic chest pain. She was discharged 10 days prior, after IR drainage of the hepatic abscess and completed 14 days of levofloxacin and metronidazole. No recent travel, sick contact, and oral contraceptive use were reported. She is allergic to sulfa and Depakote (hives). She smokes four cigarettes/day from age 21, drinks no alcohol, lives in a shelter for the past 20 years, and reported former intravenous (IV) heroin use (last use was one year ago). She has a significant past medical history of chronic hepatitis B that was diagnosed 20 years ago, was previously on tenofovir 3 years ago, had chronic hepatitis C diagnosed in 2015, and was treatment naive. On palpation, mild tender hepatomegaly was noticed and other general and physical examinations were unremarkable, except for a sternotomy scar and a mid-vertical laparotomy scar. Complete blood count showed leukocytosis of 17.1 with a left shift and liver enzymes within the normal limit. Aspirated fluid cultures from the abscess showed Providencia susceptible to amikacin, aztreonam, cefepime, cefotetan, imipenem, and Bactrim. Blood and urine cultures were negative. The HIV test was negative. A hepatitis B virus (HBV) deoxyribonucleic (DNA) polymerase chain reaction (PCR) showed > 17 million IU/ml and HCV Ab reactive high s/co ratio. A right upper quadrant sonogram showed 4.1x0.9x2.7 cm cystic formation/well-defined fluid collection in the right upper abdomen anterior to the right liver lobe (Figure 1). A computed tomography (CT) of the abdomen showed a dominant 5.2x5.5x3.9 cm hypodense lesion consistent with an abscess above the right liver (Figure 2). Initially, the patient was managed empirically with IV antibiotics, piperacillin-tazobactam, and anticoagulation since all available clinical parameters were highly in favor of pyogenic liver abscess (PLA). Clinical and laboratory improvement were achieved with intravenous antibiotics. The patient was discharged with continuing antibiotics for a total of four weeks. After four weeks of antibiotics, the repeated CT abdomen showed complete resolving of the previously seen small collection/cyst anterior to the liver dome since the previous exam (Figure 3) and she was recommended to follow up with the infectious disease (ID) clinic for chronic hepatitis B treatment, as she is noncompliant with tenofovir, and for chronic hepatitis C treatment.

## Discussion

P. stuartii is commonly reported among Providencia species to cause human infections, and their antibiotic resistance is remarkable: 82% with amoxicillin-clavulanate, 40% with ampicillin-sulbactam, 80% with gentamicin, and 84% with ciprofloxacin [3]. In a 12-year study in Ohio Hospital, P. stuartii was the source of bacteremia in 49 patients [3], where 78% of the cases are above 70 years, 96% are from a nursing home, while 92% have chronic Foley catheters on admission. The incidence is rising and found in 54% of urine samples (74% of elderly) collected from indwelling or condom catheters of bedridden patients [4].

In the US, the incidence is 3.6 cases in 100,000 individuals annually and much higher in Taiwan [5]. The most presenting symptoms from hepatic abscess are fever with or without chills and tenderness or pain in the right upper abdomen, associated with an elevated white cell count, a low albumin level, and high serum alkaline phosphatase [6]. Complications include secondary infections to the chest, peritonitis from rupture of the abscess, the Budd-Chiari syndrome secondary to compression of the hepatic vein, or inflammation of nearby structures.

The common causal agents in the western world are Escherichia coli, Klebsiella pneumoniae, and Enterococcus, in sequence. The common site is the hepatobiliary system while penetrating wounds are the second. A CT scan and a right upper quadrant (RUQ) sonogram are diagnostic tools while aspiration, staining, and culture of abscess material (aerobic and anaerobic) can get the pathological diagnosis.

Abscesses with diameters of five cm and less are managed with either needle aspiration or percutaneous catheter drainage for seven days. However, abscesses greater than five cm in diameter are best drained with a percutaneous catheter [7]. Responders to drainage are treated for two-four weeks with parenteral antibiotics but for four-six weeks if the response is unfavorable.

Carbapenems are empirically used in life-threatening situations while amikacin and piperacillin-tazobactam are used in less severe infections [8]. The choice of empirical oral antibiotics includes amoxicillin-clavulanate alone or fluoroquinolone plus metronidazole. Antibiotics should be tailored according to culture and sensitivity results later in the course [9].

## Conclusions

P. stuartii induced PLAs are extremely rare. However, in our patient, long-term shelter residence, intravenous heroin use, and chronic hepatitis (both B and C) might be precipitating factors for blood spread from the skin to cause PLA. The management of a recurrent primary hepatic abscess caused by P. stuartii is similar to a PLA from other bacterial causes: drainage and antibiotic therapy. Although in our case, the recurrent hepatic abscess is larger than five cm, the patient responded well to medical treatment according to aspirated culture sensitivity and resistivity without repeated surgical drainage.
